# Overview of Mucormycosis Cases in Türkiye: A Cumulative Case Analysis

**DOI:** 10.3390/jof12060443

**Published:** 2026-06-17

**Authors:** Melike Yaşar Duman, Egemen Bolat, Seyfi Durmaz, Dilek Yeşim Metin, Süleyha Hilmioğlu-Polat

**Affiliations:** 1 Department of Medical Microbiology, Faculty of Medicine, Ege University, İzmir 35100, Türkiye; egemen.bolat@ege.edu.tr (E.B.); dilek.metin@ege.edu.tr (D.Y.M.); suleyha56@gmail.com (S.H.-P.); 2 Department of Employee Health and Safety, Faculty of Medicine, Ege University, İzmir 35100, Türkiye; seyfi123@gmail.com

**Keywords:** mucormycosis, *Mucorales*, invasive fungal infections, Türkiye, cumulative case analysis

## Abstract

Background: Mucormycosis is a life-threatening angioinvasive fungal infection that primarily affects immunocompromised individuals, especially those with diabetes mellitus or hematologic malignancies. Despite its clinical importance, a comprehensive synthesis of mucormycosis cases from Türkiye is lacking. This study aimed to review and synthesize the published case reports on mucormycosis in Türkiye systematically. Materials and Methods: Mucormycosis case reports from Türkiye published between 1 January 1966 and 30 November 2024 were reviewed and analyzed, with an emphasis on demographics, clinical characteristics, treatment strategies, and outcomes. Results: A total of 199 cases from 155 publications were included. Mean age was 47.7 years, and 51.8% of patients were male. Diabetes mellitus was the most frequent underlying condition (65.8%), followed by hematologic malignancies (16.6%). Rhino-cerebral involvement was the predominant clinical form (86.4%). Histopathological confirmation was reported in 91.0% of cases, whereas microbiological confirmation was available in 47.7%. Overall mortality was 43.7%. Surgical debridement was performed in 69.3% of cases, and crude mortality was lower among patients who underwent surgery than among those who did not. Liposomal amphotericin B was the most frequently reported antifungal agent. Conclusions: This study provides a national descriptive synthesis of published mucormycosis cases from Türkiye over nearly six decades. The findings highlight the predominance of diabetes-associated rhino-cerebral disease, frequent reliance on histopathology, and the importance of combined medical–surgical management in routine practice. Because the dataset is based on heterogeneous published case reports and case series spanning a long time period, treatment-outcome findings should be interpreted descriptively.

## 1. Introduction

Mucormycosis is a life-threatening fungal infection caused by filamentous mold of the order *Mucorales*, characterized by angioinvasion, thrombosis, and tissue necrosis. It affects immunocompromised individuals, including those with hematologic malignancies, hematopoietic stem cell or solid organ transplants, and diabetes mellitus. Other risk factors include metabolic acidosis, and therapies involving corticosteroids, immunosuppressive agents or deferoxamine, malnutrition, prematurity, and trauma [1,2].

Mucormycosis has been associated with a mortality rate as high as 50% [3]. The COVID-19 pandemic led to an alarming increase in mucormycosis cases, particularly in India, where the condition was termed COVID-19-associated mucormycosis (CAM). While multicenter studies such as COSMIC (n = 2826) and MUCOVI (n = 1733) reported mortality rates of 14% and 32%, respectively [4,5], some smaller studies showed rates as low as 7% in certain patient populations, often due to early diagnosis and surgical debridement [6]. In Türkiye, a multicenter study reported a mortality rate of 38% among CAM patients [7]. There were additional challenges during the pandemic period such as drug shortages and delays in diagnosis and treatment [8].

Despite the rising global and regional prevalence of mucormycosis [9], Türkiye lacks a comprehensive national-level review of case data. Türkiye is located at the intersection of Europe and Asia, and country-level data from such a transition region may provide valuable context for future regional and international comparisons. Moreover, individual case reports, small case series, and locally indexed publications may be underrepresented in broader international reviews. Therefore, this study aimed to systematically compile and analyze mucormycosis cases reported in Türkiye between 1966 and 2024, encompassing a broad historical period. Through this retrospective cumulative case analysis, we evaluated patient demographics, clinical presentations, diagnostic approaches, treatment strategies, and outcomes, with the aim of identifying national patterns, addressing an important evidence gap, and providing a standardized dataset that may support future systematic reviews, surveillance efforts, and studies incorporating molecular identification and antifungal susceptibility data.

## 2. Materials and Methods

### 2.1. Study Design

This study was designed as a retrospective cumulative case analysis to analyze data on mucormycosis cases reported in Türkiye. The analysis incorporated a systematic review of published case reports between 1 January 1966 and 30 November 2024, following the PRISMA (Preferred Reporting Items for Systematic Reviews and Meta-Analyses) guidelines [10]. The study aimed to evaluate these reports’ demographic, clinical, and treatment-related data to comprehensively understand mucormycosis epidemiology and clinical patterns within the Turkish context. The methodology included a structured and systematic literature search alongside secondary data analysis, ensuring the consistency and reliability of findings in line with international practices for systematic reviews.

### 2.2. Search Strategy

A systematic literature search was conducted using PubMed, Web of Science Core Collection, and TR Dizin databases to identify eligible studies. Search terms included combinations of the keywords “mucormycosis,” “mucor,” “zygomycosis,” “case reports,” and “Türkiye.” To complement this, supplementary data were sourced from the book “Türkiye Klinik Mikoloji ve Mantar Hastalıkları Kaynakçası (Turkish Clinical Mycology and Fungal Diseases Bibliography), 1896–2004 (Yerli Yayınlar-Local publications)” and additional materials available in the library database [11]. The PubMed search strategy was optimized using Medical Subject Headings (MeSH) terms and title/abstract keywords to ensure comprehensive retrieval of relevant articles. The search criteria included “mucormycosis,” “mucor,” “zygomycosis,” “case reports,” and “Türkiye.” As MeSH terms are not supported in the Web of Science and TR Dizin platforms, searches in these databases were conducted using subject headings, titles, and abstracts to identify eligible records. This multi-source and tailored approach ensured a thorough and systematic identification of relevant studies for the analysis. In addition to the PRISMA framework for reporting systematic review, we used the CARE (Case Report) checklist, a validated tool, to assess the internal quality, reporting transparency and ethical compliance of individual case reports. Using CARE enabled us to evaluate the methodological rigor of reports in a standardized way and identify underreported components, such as ethical consent and patient perspective.

### 2.3. Inclusion and Exclusion Criteria

The inclusion criteria for this study encompassed case reports published between 1966 and 2024, focusing on mucormycosis-related infections with keywords such as “*mucormycosis*,” “*mucor*,” “*zygomycosis*,” and “*case reports*.” Only studies conducted in Türkiye and involving human subjects were considered eligible. Furthermore, the selected reports were required to provide definitive microbiological or histopathological evidence confirming the diagnosis of mucormycosis.

Only studies in the format of individual case reports were included in this review. Reports presenting long case series without a case report format or without individual-level clinical data were excluded. The inclusion focused strictly on articles detailing one or a few cases with comprehensive information regarding clinical presentation, diagnostics, treatment, and outcomes. Therefore, large-scale case series or cohort studies, even if conducted in Türkiye, were not eligible unless they reported cases in a structured case report format.

Studies were excluded if they represented duplicate records or were not categorized as case reports, including projects, conference abstracts, or general research articles. Non-human studies and reports from geographic locations outside Türkiye were also excluded to maintain the study’s contextual relevance. Additionally, case reports focusing on infections caused by fungal agents other than mucormycosis, as well as those lacking accessible abstracts or full texts, were omitted. Reports without microbiological or histopathological evidence confirming the diagnosis or those not explicitly classified as infections were similarly excluded from upholding methodological rigor and ensuring the validity of the analysis. This structured approach allowed for the systematic inclusion of high-quality and contextually relevant data.

### 2.4. Study Selection

The final search results from each database were imported into Excel, and duplicates were removed. The remaining articles underwent title and abstract screening based on the eligibility criteria, and no reasons were given for excluding articles at this stage. The full text was then screened using the CARE checklist. Eligibility for inclusion was determined, and reasons for excluding articles were recorded. Two researchers (MYD and EB) performed title/abstract screening and full-text screening independently. A third reviewer (DYM) resolved all disagreements.

To describe reporting completeness of the included case reports, selected CARE checklist items were assessed and summarized descriptively in the Supplementary Table S1 [12].

Each criterion was assessed as “sufficient,” “not sufficient,” or “partially sufficient,” depending on the content of the case report. A response of “sufficient” indicated that the criterion was fully met; “partially sufficient” indicated that the criterion was partially addressed; and “not sufficient” indicated that the criterion was not included in the report. Special attention was given to the presence of ethical approval and informed consent statements during the evaluation process. The results obtained aimed to determine the methodological and ethical compliance level of the case reports included in the review. The scientific quality and publication readiness of the case reports could be determined using this approach.

Among the case reports evaluated, six case reports in a letter-to-the-editor format scored high on the CARE checklist criteria and were included in the dataset to increase inclusiveness.

### 2.5. Data Extraction

Data extraction was performed using a standardized data collection form to ensure consistency and accuracy across all included case reports. The extracted data encompassed demographic, clinical, treatment, and outcome variables critical to the analysis.

Demographic Data: Patient age and gender were systematically recorded.

Clinical Features: Data on the referred department, patient comorbidities, presenting symptoms, clinical findings, and affected anatomical areas were documented. Case classification was based on the definitions of the Centres for Disease Control and Prevention (CDC). Mucormycosis cases were classified as rhino-cerebral, pulmonary, cutaneous, gastrointestinal, disseminated, and other localized forms of mucormycosis [1]. Terms such as rhino-orbito-cerebral, orbital, and rhino-cerebral mucormycosis have been grouped under the category of rhino-cerebral mucormycosis. Cases of urinary and cardiac mucormycosis were classified as other localized forms of mucormycosis, while cases of liver mucormycosis were classified as gastrointestinal mucormycosis. Additionally, results from radiological imaging (CT, MRI, and radiography), microbiological examinations (direct microscopy and culture results), and histopathological analyses were included to confirm diagnosis and characterize disease presentation.

Treatment Data: Details on antifungal therapy, including its intent (empirical, curative, or prophylactic) and the specific antifungal agents used, were extracted. Only patients who underwent debridement for disease control were recorded as having undergone surgery. Surgery was not considered to include biopsies performed to document the disease.

Outcomes: Patient outcomes, particularly mortality and survival, were meticulously documented to evaluate treatment efficacy and overall prognosis.

### 2.6. Statistical Analysis

All statistical analyses were conducted to evaluate the extracted data systematically and derive meaningful insights from the case reports. Descriptive statistics were used to summarize demographic, clinical, treatment, and outcome variables, including frequencies, percentages, means, and standard deviations. Continuous variables such as age were presented as means with standard deviations, while categorical variables, including gender and clinical features, were reported as frequencies and percentages.

Given the systematic review design, variability among the included single case reports and case series was explored primarily through descriptive analysis, focusing on clinical presentations, treatment modalities, and patient outcomes. The inherent heterogeneity of the data was addressed by identifying common patterns and significant differences within the reported variables.

The included studies’ methodological diversity was evaluated using established quality assessment tools such as the CARE guidelines to enhance the analysis’s robustness. These frameworks ensured a structured appraisal of the case reports’ consistency, completeness, and transparency.

Exploratory inferential analyses were conducted to assess potential associations between variables. Correlation analyses were used to evaluate relationships between demographic factors (e.g., age, gender) and outcomes (e.g., survival status). Logistic regression models were constructed based on variables showing significance in univariable analyses and clinical relevance. Odds ratios (ORs) with 95% confidence intervals (CIs) were calculated to estimate the strength of associations. Due to limited sample size and missing data, only a restricted number of variables were included in multivariable models to avoid overfitting. Given the cumulative case-report design and data heterogeneity, multivariable analyses were performed in an exploratory manner, and no causal inference was intended.

Data analysis was performed using SPSS Statistics software (version 25.0, IBM Corp., Armonk, NY, USA) to ensure the precision and reliability of the results. This rigorous statistical approach facilitated a robust interpretation of the data, providing comprehensive insights into the epidemiological and clinical characteristics of mucormycosis cases.

## 3. Results

**Figure 1:**
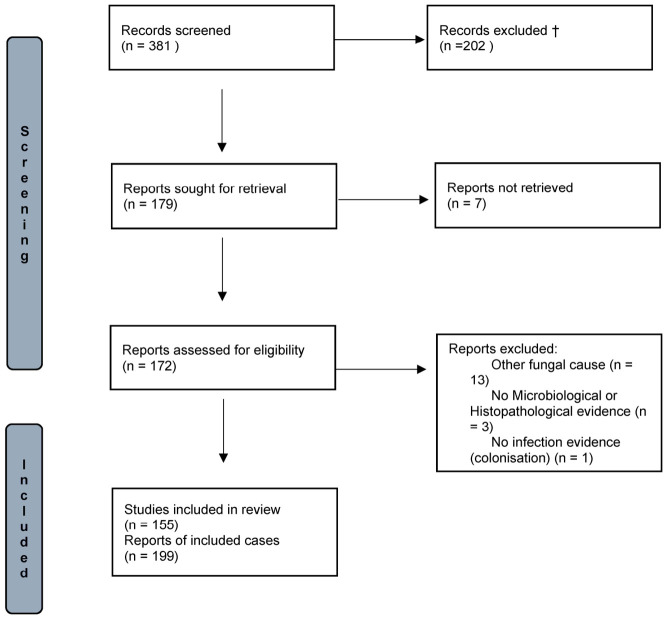
Flow diagram for selection of studies included in the systematic review. The PRISMA Statement. † Duplication (n = 69), and non-relevant (n = 133).

A total of 381 articles published between 1 January 1966 and 30 November 2024 were identified through databases and library searches. After removing duplicates and irrelevant records, 155 articles that met the inclusion criteria were included in the final analysis (Figure 1).

Reporting completeness was generally high for core clinical and diagnostic items, whereas ethical approval/informed consent statements and patient perspective were infrequently reported (Supplementary Table S1).

### 3.1. Study Characteristics

The dataset, totaling 199 cases, comprised primarily single case reports (81.3%, n = 126), supplemented by small case series (18.7%, n = 29). This approach enabled the accumulation of rare cases, specifically including one report of six, two reports of four, seven reports of three, and 19 reports of two cases. Of the cases, 179 (89.9%) belonged to the years 1996 and after, which is the year of the introduction of Liposomal Amphotericin B in Türkiye [13].

### 3.2. Demographic and Clinical Characteristics

Across the 199 patients identified through the systematic review, the mean age was 47.7 ± 19.5 years (Range: 0–84). Male patients comprised 52.3% of the cases, yielding a male-to-female ratio of 1:1.

Clinical presentation was primarily analyzed according to the anatomical site of involvement rather than individual symptoms. Rhino-cerebral mucormycosis was the predominant clinical form, accounting for 86.4% of all cases, followed by pulmonary (5.5%), gastrointestinal (3.5%), cutaneous (2.5%), and other localized forms (2%). Because clinical symptoms are inherently determined by the anatomical site of infection, individual symptoms such as headache, fever, hemiparesis, or altered mental status were not analyzed separately in the main text. These findings did not provide additional analytical value beyond disease localization and were therefore removed to improve readability (Table 1).

Chronic underlying diseases were present in 89.9% of cases, most commonly diabetes mellitus, followed by malignancies—hematological—chronic kidney disease, immunodeficiency, and solid organ transplantation.

A prevalence of chronic diseases was observed in 89.9% of the mucormycosis cases. The most reported comorbidities were diabetes mellitus (65.8%), malignancies (20.1%), the majority (16.6%) of which are hematological malignancies, chronic kidney disease (9.5%), immunodeficiency (8.0%), and solid organ transplantation (6.5%, including kidney transplantation in 5.5% and liver transplantation in 1.0%).

Furthermore, of the 199 patients, 177 underwent radiological imaging, and 140 had findings on a computed tomography scan.

### 3.3. Microbiological Examination

**Figure 2:**
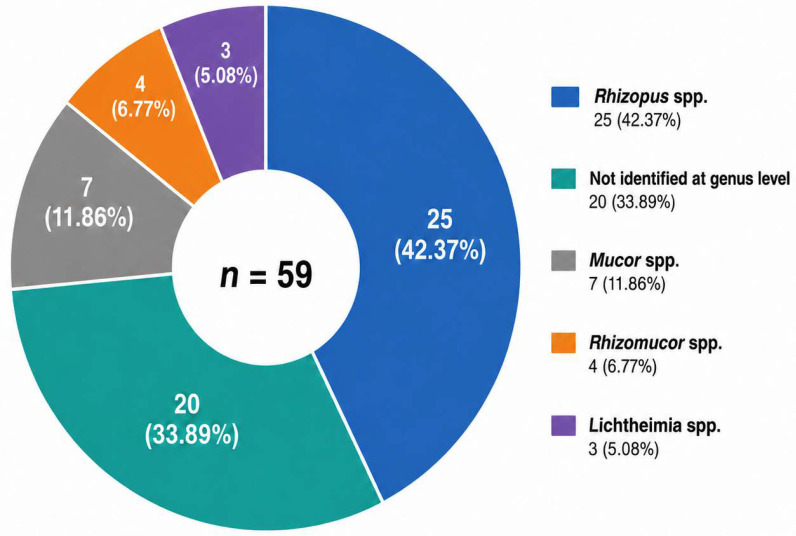
Distribution of culture-positive Mucorales isolates reported from Türkiye (n = 59). Isolates originally reported as *Absidia corymbifera* were harmonized according to current taxonomy and are presented under *Lichtheimia* spp. The “not identified at genus level” category includes culture-positive cases in which the original publication reported the isolate only as Mucorales, Zygomycetes/Phycomycetes, or otherwise did not provide genus-level identification. *Rhizopus* spp. accounted for 25/59 isolates (42.37%) and included *Rhizopus arrhizus* (12/59, 20.34%); the remaining *Rhizopus* isolates were reported only at the genus level. *Mucor* spp. accounted for 7/59 isolates (11.86%) and included *Mucor circinelloides* (2/59, 3.39%).

Out of the 199 cases analyzed, 95 (47.7%) had reported microbiological evidence, defined as positive direct microscopy and/or culture supporting the diagnosis of mucormycosis. The remaining 104 cases (52.3%) had no reported microbiological evidence in the original case reports. This category should not be interpreted as culture negativity, because many historical reports did not clearly state whether microbiological testing was not performed, yielded negative results, or was simply not reported in sufficient detail. In 36 of the 95 cases with microbiological evidence, hyphal structures were observed on direct microscopy, whereas in 59 cases these findings were not reported. In addition, 73 of these 95 cases (76.8%) showed fungal growth in culture, while seven cases (7.4%) showed no fungal growth. Missing data on culture results were reported in 15 cases (7.5%). In the case reported in 1966, the growing fungus was defined as One culture-positive isolate was reported in the original publication as Phycomycetes. As no genus-level identification was provided, this isolate was classified under the “not identified at genus level” category in Figure 2.

### 3.4. Histopathological Evidence

Histopathological findings were reported in 188 cases (94.5%), with 171 cases (91%) showing definitive histopathological evidence consistent with mucormycosis. Conversely, 17 cases (9%) lacked histopathological evidence, and data was missing for 11 cases (5.5%).

### 3.5. Treatment Modalities

Surgical Debridement: Out of the 199 cases analyzed, 138 (69.3%) involved surgical debridement as part of the treatment strategy, while 61 (30.7%) did not include surgical intervention.

Antifungal Treatment: Antifungal treatments were categorized as prophylactic, empiric, or curative. Five patients were reported to be receiving antifungal prophylaxis at the time of mucormycosis diagnosis (fluconazole, n = 3; amphotericin B, n = 2). Underlying conditions included hematologic malignancy (n = 3), solid organ transplantation (n = 1), and aplastic anemia (n = 1). In addition, empiric antifungal treatment was identified in 101 cases. Among the antifungals used in empirical treatment, LAMB was reported in 48 cases (47.52%), amphotericin B in 45 cases (44.55%), voriconazole in 6 cases (5.94%), fluconazole in 3 cases (2.97%), caspofungin in 3 cases (2.97%), and ketoconazole in 1 case (0.99%).

Curative antifungal treatment data were available for 180 cases (91.83%), while treatment details were missing in 19 cases (9.54%). Among the reported cases: LAMB in 100 cases (57.47%), amphotericin B in 86 cases (49.43%), posaconazole in 28 cases (16.09%), fluconazole in 5 cases (2.87%), itraconazole in 3 cases (1.72%), ketoconazole in 1 case (0.57%).

Among the 199 cases analyzed, survival outcomes were documented for all patients. Of these, 109 cases (54.8%) resulted in survival, while 87 (43.7%) concluded with mortality. Additionally, 3 cases (1.5%) had inconclusive outcomes.

### 3.6. Comparison of Diagnostic Methods

Histopathological and microbiological findings, as well as their associations with imaging modalities and clinical variables, are summarized in Table 2.

A statistically significant association was observed between histopathological findings and microbiological examination results (*p* < 0.001). However, agreement between the two methods was poor, with a negative kappa coefficient.

No statistically significant association or agreement was found between microbiological examination results and MRI, CT, or radiographic findings. Similarly, microbiological results were not significantly associated with the presence of symptoms or clinical findings.

### 3.7. Subgroup Analyses

Among the 195 cases with valid data on survival and gender, survival rates were comparable between male and female patients. Of the 93 female patients, 50 (53.8%) survived, while 43 (46.2%) did not. Similarly, among the 102 male patients, 59 (57.8%) survived, and 43 (42.2%) succumbed to the disease (*p* = 0.567) (Table 3). The association between major comorbidities and mortality was additionally analyzed using the chi-square test of independence. Diabetes mellitus was not significantly associated with mortality (OR: 1.42, 95% CI: 0.78–2.56; *p* = 0.249). Similarly, hematological malignancy was not significantly associated with mortality in this cohort (OR: 0.53, 95% CI: 0.25–1.12; *p* = 0.095).

### 3.8. Symptoms and Mortality

Certain neurological symptoms, including mental confusion and hemiparesis, were associated with higher mortality in univariable analyses. Detailed symptom-based mortality data are provided Table 3.

### 3.9. Treatment and Mortality

The logistic regression analysis evaluated the association between treatment modalities and mortality, both before and after adjusting for potential confounders. The results are summarized below Table 4.

Surgical Debridement: Surgical debridement showed a significant relationship with mortality (*p* < 0.001). Among patients who did not undergo surgical debridement, the mortality rate was 73.3%, compared to 31.6% in those who underwent surgical intervention. Surgical debridement showed a strong protective effect against mortality, with an unadjusted OR of 0.168 (95% CI: 0.085–0.331, *p* < 0.001). After adjustment, this association remained highly significant, with an adjusted OR of 0.180 (95% CI: 0.090–0.362, *p* < 0.001).

**Antifungal Therapy:** Treatment regimens were categorized as amphotericin B alone, LAMB alone, or combined therapy. Combined therapy demonstrated the lowest mortality rate (26.9%), followed by LAMB alone (43.3%) and amphotericin B alone (49.2%) (*p* = 0.046).

Posaconazole use: The use of posaconazole was significantly associated with reduced mortality (*p* < 0.001). Patients treated with posaconazole had a mortality rate of 48.6%, compared to 85.2% of those who did not receive this therapy. In the unadjusted model, the use of posaconazole was significantly associated with reduced mortality, with an odds ratio (OR) of 0.183 (95% CI: 0.060–0.551, *p* = 0.003). After adjustment, the association remained significant, with an adjusted OR of 0.245 (95% CI: 0.075–0.794, *p* = 0.019).

Liposomal Amphotericin B (LAMB) use: The use of LAMB was also significantly related to mortality outcomes (*p* = 0.013). Patients receiving LAMB had a mortality rate of 35.4%, compared to 53.2% of those who did not receive it. The use of LAMB was significantly associated with lower mortality in the unadjusted model, with an OR of 0.481 (95% CI: 0.270–0.858, *p* = 0.013). However, this association was no longer statistically significant after adjustment, with an adjusted OR of 0.667 (95% CI: 0.350–1.271, *p* = 0.219).

Amphotericin B use: The relationship between amphotericin B use and mortality was evaluated in 196 patients. Among those who did not receive amphotericin B, the mortality rate was 40.7% (44 out of 108), while the survival rate was 59.3% (64 out of 108). In contrast, among patients who received amphotericin B, the mortality rate was slightly higher at 48.2% (41 out of 85), with a survival rate of 51.8% (44 out of 85). However, the difference in mortality between the two groups was not statistically significant (*p* = 0.298). Amphotericin B use was not significantly associated with mortality in the unadjusted model (OR: 1.355, 95% CI: 0.764–2.404, *p* = 0.298) (Table 4).

## 4. Discussion

**Figure 3:**
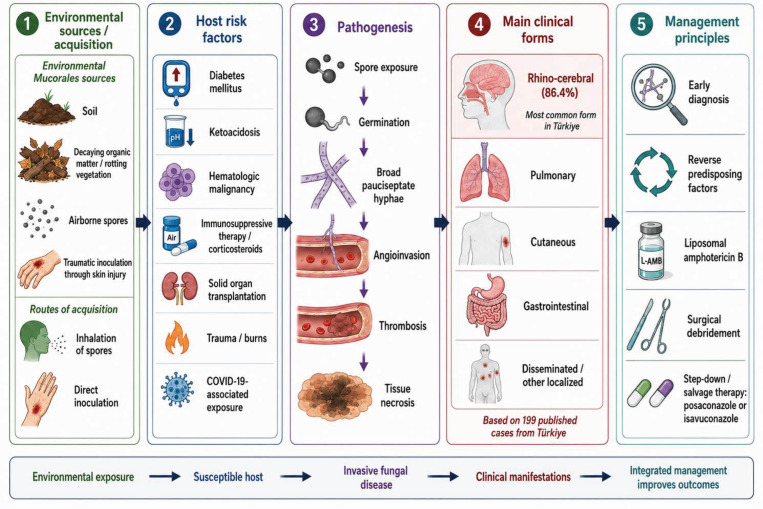
Schematic overview of mucormycosis pathogenesis, clinical presentation, and management. Mucormycosis is acquired mainly through inhalation of environmental Mucorales spores or direct traumatic inoculation. In susceptible hosts, including patients with diabetes mellitus, hematologic malignancy, transplantation, immunosuppressive treatment, or trauma, spores may germinate into broad, pauciseptate hyphae. Angioinvasion leads to vascular thrombosis, tissue ischemia, and necrosis. The main clinical forms include rhino-cerebral, pulmonary, cutaneous, gastrointestinal, disseminated, and other localized diseases. Management relies on early diagnosis, reversal of predisposing factors, when possible, prompt antifungal therapy, and surgical debridement when indicated.

Mucormycosis is a rare but severe angioinvasive fungal infection caused by fungi of the order Mucorales and is associated with substantial mortality, particularly among immunocompromised individuals [1]. In this systematic review of cases reported from Türkiye, we identified distinct clinical and epidemiological patterns, including a predominance of diabetes-associated rhino-cerebral disease. The findings also highlight key diagnostic and management challenges that reflect real-world clinical practice. A schematic overview of the environmental acquisition, host risk factors, pathogenesis, clinical forms, and management principles of mucormycosis is provided in Figure 3.

In contrast to large cohort studies (e.g., COSMIC and MUCOVI) reporting male predominance, our analysis showed a nearly equal sex distribution [4,5]. Given the case-report–based nature of the data, this finding should be interpreted cautiously and may reflect reporting bias rather than true epidemiological differences. Overall, these findings reflect the demographic and clinical profile of mucormycosis cases reported from Türkiye, with a predominance of rhino-cerebral disease and underlying metabolic or immunosuppressive conditions.

## 5. Comorbidities

Mucormycosis predominantly affects patients with underlying diseases or impaired host defenses. Established risk factors include diabetes mellitus, particularly diabetic ketoacidosis, hematological malignancy, solid organ transplantation, neutropenia, prolonged corticosteroid use, iron overload, HIV infection, trauma, and exposure to immunomodulating drugs, including voriconazole in selected cases [167]. In the present study, chronic underlying diseases were reported in most patients (89.9%), supporting the central role of host-related factors in the development of mucormycosis. Diabetes mellitus was the most common comorbidity (65.8%), followed by hematological malignancy (16.6%) and chronic kidney disease (9.5%). Similar diabetes predominance has been reported in India, Iran, Taiwan, and Mexico. In contrast, hematological malignancy is more frequently the leading underlying condition in several European, North American, and other international series [9,168,169,170,171,172,173]. Thus, our findings are consistent with the literature showing that diabetes mellitus and hematological malignancy are among the major predisposing conditions for mucormycosis.

The high proportion of diabetes in our dataset may partly explain the predominance of rhino-cerebral mucormycosis in Türkiye. This observation is also compatible with the high diabetes burden reported globally and nationally. According to the World Health Organization, the number of people living with diabetes has increased markedly worldwide, with a particularly rapid rise in low- and middle-income countries [174]. The IDF 2024 report estimated that 9.6 million adults in Türkiye were living with diabetes, corresponding to a prevalence of 16.3%, with 45.5% of cases remaining undiagnosed [175]. However, because the present analysis was based on retrospective and heterogeneous published case reports, this data cannot be used for formal risk estimation.

## 6. Clinical Forms of Mucormycosis

Mucormycosis presents in a variety of forms depending on the organ involved. These include rhinocerebral, pulmonary, cutaneous, gastrointestinal, or disseminated infections [1]. In our study, rhinocerebral involvement was reported in 86.4% of cases. Pulmonary involvement was seen in 5.5%, gastrointestinal involvement in 3.5%, cutaneous mucormycosis in 2.5% and other localized forms of mucormycosis in 2%. According to the CDC, rhinocerebral mucormycosis is most common in patients with diabetes and kidney transplant recipients [1]. In contrast, in France, where hematological malignancies are the predominant underlying disease, the most common form of mucormycosis is pulmonary mucormycosis (52.4%) [172]. The proportion of rhinocerebral mucormycosis was remarkably high in our analysis. In this systematic review, diabetes was the most common co-morbidity, and rhinocerebral mucormycosis was the most common form of mucormycosis in all patients. In addition, rhinocerebral mucormycosis was found to be the most common form in 81.8% of patients with hematological malignancies.

Mucormycosis is a fungal infection that can be seen in a variety of clinics and even emergency departments, depending on the clinical presentation. In patients with risk factors such as hematological malignancy, immunosuppression, uncontrolled diabetes and COVID-19, rhinocerebral mucormycosis should be considered in the differential diagnosis in the presence of non-specific symptoms such as fever, headache, postnasal discharge and sinonasal congestion [21,176]. In this study, it was found that surgical units (16.08%) conducted the most frequent evaluations, and these units were particularly concentrated in the fields of otolaryngology (9.55%) and ophthalmology (5.03%). The rate of cases evaluated in internal medicine units was 10.05%, and almost half of these cases were evaluated in hematology units. The rate of emergency department use of 9.55% underscores the importance of acute care units in diagnosing and managing these patients. Most of the patients admitted were evaluated as rhino-cerebral mucormycosis. Early diagnosis is essential to prevent the spread of infection and improve clinical outcomes.

## 7. Diagnostic Methods

A microbiological examination is essential for the etiological diagnosis of mucormycosis and identification of the causative agent. In the present analysis, 95 of 199 cases (47.7%) had microbiological evidence, defined as positive direct microscopy and/or culture, whereas 104 cases (52.3%) lacked reported microbiological evidence. Among cases with microbiological evidence, hyphal structures were observed by direct microscopy in 36 cases (37.9%), and fungal growth was reported in culture in 73 cases (76.8%). Although culture is known to have limited sensitivity in mucormycosis and failure to isolate the pathogen is common, culture-positive cases remain important because they allow genus- or species-level identification and may provide epidemiological information.

In culture-positive cases, *Rhizopus* spp. was the most frequently reported genus, accounting for 42.2% of culture-positive isolates, with *Rhizopus arrhizus* identified in 20.3% of cases (Figure 2). The remaining *Rhizopus* isolates were reported only at the genus level in the original publications; therefore, species-level assignment could not be inferred. *Mucor* spp. and *Lichtheimia* spp. were less commonly reported. Because most historical reports relied on conventional culture and morphology rather than molecular identification, species-level resolution was limited. These findings should therefore be interpreted as a genus/species-level distribution of reported culture-positive Mucorales isolates rather than a complete species-level epidemiological analysis.

Histopathological findings were reported in 188 cases (94.5%), of which 171 cases (91.0%) had histopathological evidence consistent with mucormycosis. Histopathology remains a cornerstone of diagnosis because it demonstrates characteristic fungal morphology and tissue invasion. However, it does not allow species-level identification or antifungal susceptibility testing. Conversely, microbiological methods support etiological identification but may be limited by culture negativity and incomplete reporting. In our analysis, histopathological findings and microbiological examination results were significantly associated (*p* < 0.001), but agreement between these two diagnostic modalities was poor, as reflected by the kappa coefficient. This finding supports the need for an integrated diagnostic approach rather than reliance on a single method. Therefore, the combined use of histopathology and microbiology is considered the gold standard for the definitive diagnosis of mucormycosis [177].

Molecular methods may further support the diagnosis of mucormycosis, particularly in culture-negative cases, in patients who have already received antifungal therapy, or when only limited tissue material is available. Mucorales-specific PCR assays and sequencing-based approaches can be applied to fresh tissue, formalin-fixed paraffin-embedded tissue, serum/plasma, or bronchoalveolar lavage fluid, depending on the method and clinical context. These techniques may contribute to earlier detection, species-level identification, recognition of mixed fungal infections, and epidemiological surveillance. However, molecular diagnosis of mucormycosis has not yet been universally standardized and is not currently established as a stand-alone definitive diagnostic criterion in major consensus definitions. In the present cumulative case analysis, molecular testing was not reported in the historical case reports; therefore, its diagnostic yield could not be systematically evaluated.

No significant association was observed between microbiological findings and imaging modalities. This result reflects the distinct diagnostic roles of these methods rather than a lack of clinical relevance. CT and MRI are essential for assessing disease extension, anatomical involvement, and surgical planning, whereas microbiological and histopathological methods are required to confirm the underlying etiology. Therefore, imaging should be interpreted as complementary to, but not a substitute for, etiological confirmation in mucormycosis [167].

Similarly, no significant relationship was observed between microbiological results and clinical findings (*p* = 0.899) or symptoms (*p* = 0.324). This is clinically expected because mucormycosis often presents with non-specific symptoms and may mimic other infectious or inflammatory conditions. Accurate diagnosis therefore requires integration of clinical suspicion, imaging findings, histopathological evidence, and microbiological confirmation whenever available.

## 8. Treatment Approaches and Impact on Mortality

In this review, data extraction focused on treatment-related parameters, including the use of antifungal therapy, the agents administered, and whether surgical debridement was performed. Antifungal susceptibility testing was not systematically assessed because such information was rarely reported in the included case reports; therefore, susceptibility patterns and their potential impact on treatment outcomes could not be evaluated. Antifungal treatments were classified as prophylactic, empiric, or curative based on clinical indication. However, given the retrospective and descriptive nature of the data and the possibility of confounding by indication, treatment categories were not interpreted as independent predictors of mortality.

Management of mucormycosis requires early diagnosis, prompt antifungal therapy, reversal of underlying predisposing factors when possible, and surgical debridement when indicated. Liposomal amphotericin B (LAMB) is recommended as a first-line agent for invasive mucormycosis in international guidelines because of its efficacy and more favorable toxicity profile compared with conventional amphotericin B formulations [2]. In the present analysis, curative antifungal treatment data were available for 180 cases, and the most frequently reported agents were LAMB, amphotericin B, and posaconazole. This pattern is consistent with current guideline-based management principles; however, detailed information on dose, timing, duration, and treatment indication was inconsistently reported across the historical case reports.

In unadjusted analyses, LAMB use was associated with lower mortality; however, this association was no longer statistically significant after adjustment. Amphotericin B use was not significantly associated with mortality. The apparent association between posaconazole use and lower mortality should also be interpreted cautiously, because posaconazole became available during the later years of the study period and was more likely to be used in selected patients, including those who survived initial therapy or had less severe disease. Therefore, these antifungal-related findings should be regarded as descriptive associations rather than evidence of independent treatment effects.

Surgical debridement showed the most consistent association with improved survival. In our dataset, debridement was performed in 69.3% of cases, and mortality was significantly lower among patients who underwent surgery than among those who did not. This association remained significant after adjustment, supporting the importance of combined medical–surgical management when feasible. In comparison, Manesh et al. reported surgical treatment in 65.2% of 184 patients, although the rate was substantially lower among patients with hematological malignancy (21.4%) [178]. These findings emphasize that timely surgical intervention remains an important component of mucormycosis management, while also highlighting that surgical feasibility may vary according to disease site, host condition, and underlying comorbidities.

## 9. Mortality Analysis

Survival status could not be determined in three cases (1.5%). Mucormycosis is generally associated with high mortality, often exceeding 50% depending on the site of involvement and host condition, and mortality may be particularly high among patients with hematological malignancies [2,179]. Previous studies have reported mortality rates of 55.8% in France and 74% in China among patients with hematological malignancies [172]. The overall mortality observed in the present study was lower than these rates; however, this difference should be interpreted cautiously because our dataset was dominated by diabetes-associated cases, hematological malignancy was present in a smaller proportion of patients, and cumulative case-report analyses may be affected by publication and survival-reporting bias.

Additional analysis showed no statistically significant association between mortality and either diabetes mellitus or hematological malignancy. Therefore, although previous studies have reported higher mortality among patients with hematological malignancies, this difference could not be demonstrated in our cumulative case-report dataset. This comparison should be interpreted cautiously because comorbidities were not mutually exclusive and subgroup sizes were limited.

Mortality rates were similar between men and women. Higher mortality was observed in cases with neurological manifestations, which are likely to reflect advanced disease with central nervous system involvement rather than symptom-specific effects. In this context, rhino-orbital disease with cranial extension, or rhino-orbito-cerebral mucormycosis, is well recognized as a major determinant of poor outcome. However, due to inconsistent reporting of radiological and neurological details across case reports, reliable stratification according to cranial extension was not feasible in this cumulative analysis. Therefore, neurological findings are interpreted as markers of disease extent rather than independent prognostic factors.

## 10. Strengths and Limitations

This study is a comprehensive review of the literature in which the published cases of mucormycosis in Türkiye between 1966 and 2024 were systematically compiled. A transparent and traceable process was followed using the PRISMA diagram, and the quality of case reports was assessed using the CARE checklist. This approach increased the study’s methodological robustness and supported the findings’ reliability. Furthermore, as such a comprehensive review of mucormycosis cases in Türkiye has not been done before, the study makes an important contribution to the local literature.

However, the study has several limitations. Firstly, the reliance on case report data alone may limit the generalizability of the findings. In addition, the heterogeneous nature of the case reports and the different writing styles of the authors made some data inaccessible and made it difficult to directly compare the data obtained. Classification of cases based on reported data only limited the scope for epidemiological inference and generalizable conclusions. Moreover, the incomplete reporting of key prognostic variables (e.g., symptom duration, antifungal dosing, timing of surgery) in many case reports restricted the depth of survival and outcome analyses. The low mortality rates may also be biased by the authors’ tendency to report more survivors.

## 11. Conclusions

This cumulative case analysis provides the first national synthesis of published mucormycosis cases from Türkiye over nearly six decades. The findings show a predominance of diabetes-associated rhino-cerebral disease, frequent reliance on histopathology, limited microbiological confirmation, and the importance of combined medical–surgical management. The heterogeneity and incomplete reporting of historical case reports limit causal interpretation of treatment–outcome associations and highlight the need for standardized diagnostic, therapeutic, microbiological, molecular, and outcome data in future reports and surveillance studies.

## Figures and Tables

**Table 1:** Demographic characteristics, symptoms, findings, clinical forms, treatment and survival status of the patients [14,15,16,17,18,19,20,21,22,23,24,25,26,27,28,29,30,31,32,33,34,35,36,37,38,39,40,41,42,43,44,45,46,47,48,49,50,51,52,53,54,55,56,57,58,59,60,61,62,63,64,65,66,67,68,69,70,71,72,73,74,75,76,77,78,79,80,81,82,83,84,85,86,87,88,89,90,91,92,93,94,95,96,97,98,99,100,101,102,103,104,105,106,107,108,109,110,111,112,113,114,115,116,117,118,119,120,121,122,123,124,125,126,127,128,129,130,131,132,133,134,135,136,137,138,139,140,141,142,143,144,145,146,147,148,149,150,151,152,153,154,155,156,157,158,159,160,161,162,163,164,165,166].

*Feature*	*Microbiological Positive n (%)*	*Microbiological Negative n (%)*	*Total n (%)*	*p Value*
**Study period**				
1966–1995	14 (13.2)	6 (6.5)	20 (10.1)	0.114
≥1996	92 (86.8)	87 (93.5)	179 (89.9)	
**Age group (years)**				
0–18	12 (11.5)	11 (11.6)	23 (11.6)	0.880
19–40	20 (19.2)	13 (13.7)	33 (16.6)	
41–60	44 (42.3)	45 (47.4)	89 (44.7)	
>60	28 (26.9)	26 (27.4)	54 (27.1)	
**Sex**				
Female	45 (42.5)	49 (53.3)	94 (47.5)	0.129
Male	61 (57.5)	43 (46.7)	104 (52.5)	
**Comorbidities/risk factors**				
Diabetes mellitus	64 (60.4)	67 (72.0)	131 (65.8)	0.083
Any malignancy	22 (20.8)	18 (19.4)	40 (20.1)	0.806
└ Hematological malignancy	18 (17.0)	15 (16.1)	33 (16.6)	NS
└ Other malignancy	3 (2.8)	3 (3.3)	6 (3.0)	
Chronic renal failure	11 (10.4)	8 (8.6)	19 (9.5)	0.671
Immunodeficiency	8 (7.6)	8 (8.6)	16 (8.1)	0.800
Immunosuppressive treatment (197)	21 (20.2)	19 (20.4)	40 (20.3)	0.967
Chemotherapy	12 (11.4)	12 (12.9)	24 (12.1)	0.751
COVID 19	5 (4.7)	6 (6.5)	11 (5.5)	0.593
Solid organ transplantation	5 (4.7)	8 (8.6)	13 (6.5)	0.268
└Renal transplantation	5 (4.7)	6 (6.5)	11 (5.5)	
└Liver transplantation	0 (0)	2 (2.2)	2 (1)	
**Symptoms**				
Symptomatic	93 (84.7)	77 (82.8)	170 (85.4)	0.324
Pain (any)	57 (53.8)	53 (57.0)	110 (55.3)	0.649
└ Facial pain	33 (31.1)	34 (36.6)	67 (33.7)	0.419
└ Headache	31 (29.3)	30 (32.3)	61 (30.7)	0.649
└ Abdominal pain	6 (5.7)	5 (5.4)	11 (5.5)	0.930
└ Eye pain	3 (2.8)	7 (7.5)	10 (5.0)	0.130
Visual impairment	51 (48.1)	40 (43.0)	91 (45.7)	0.471
Wound site/postnasal discharge	21 (19.8)	15 (16.1)	36 (18.1)	0.501
Redness and swelling of the eye	6 (5.7)	10 (10.8)	16 (8.0)	0.197
Mental convulsion	10 (9.4)	9 (9.7)	19 (9.6)	0.984
Gastrointestinal symptoms nausea/vomiting/diarrhea	13 (12.3)	9 (9.7)	22 (11.01)	0.899
**Clinical examination findings**				
Any clinical examination finding	94 (88.7)	83 (89.2)	177 (88.9)	0.899
└ Facial oedema	52 (49.1)	38 (40.9)	90 (45.2)	0.246
└ Sinonasal black lesion	58 (54.7)	55 (59.1)	113 (56.8)	0.530
└ Cranial nerve involvement	31 (29.3)	44 (47.3)	75 (37.7)	**0.009**
└ Fever	31 (29.3)	37 (39.8)	68 (34.2)	0.118
└ Ophthalmoplegia	36 (34.0)	40 (43.0)	76 (38.2)	0.190
└ Proptosis	29 (27.4)	21 (22.6)	50 (25.1)	0.438
└ Ptosis	17 (16.0)	19 (20.4)	36 (18.1)	0.422
└ Hemiparesis	19 (17.9)	17 (18.3)	36 (18.1)	0.948
└ Oral ulcer	11 (10.4)	11 (11.8)	22 (11.1)	0.745
**Diagnostic methods**				
Positive histopathological finding	106 (100)	65 (79.3)	171 (95.5)	**<0.002**
**Microbiological evaluation**				
└ Positive direct microscopy findings	36 (37.9)	-	36 (37.9) *	NA
└ Positive culture growth	73 (76.8)	-	73 (76.8) *	NA
**Radiological imaging**				
Any radiological imaging	96 (90.6)	81 (87.1)	177 (88.9)	0.436
└ Computed tomography (CT)	75 (70.8)	65 (69.9)	140 (70.4)	0.894
└ Magnetic resonance imaging (MRI)	43 (40.6)	44 (47.3)	87 (43.7)	NS
└ Direct radiography	16 (15.1)	13 (14.0)	29 (14.6)	0.824
**Clinical form**				
Rhino-cerebral mucormycosis	91 (85.9)	81 (87.1)	172 (86.4)	NS
Pulmonary mucormycosis	7 (6.6)	4 (4.3)	11 (5.5)	
Gastrointestinal mucormycosis	4 (3.8)	3 (3.2)	7 (3.5)	
Cutaneous mucormycosis	2 (1.9)	3 (3.2)	5 (2.5)	
Other localized forms of mucormycosis	2 (1.9)	2 (2.2)	4 (2.0)	
**Treatment and outcome**				
Surgical debridement	74 (69.8)	64 (68.8)	138 (69.3)	0.879
**Antifungal treatment**				
Prophylactic antifungal treatment	4 (3.8)	1 (1.1)	5 (2.5)	NS
Empiric antifungal treatment	61 (64.2)	40 (38.5)	101 (50.8)	NS
└ Liposomal amphotericin B (LAMB)	38 (36.9)	62 (66.7)	100 (51.0)	**0.001**
└ Amphotericin B	51 (49.5)	35 (37.6)	86 (43.9)	0.094
└ Posaconazole	10 (9.7)	18 (19.4)	28 (14.3)	0.054
**Curative antifungal treatment**	92 (95.8)	88 (84.6)	180 (91.8)	NS
└ Only amphotericin B	44 (50.6)	20 (22.2)	64 (36.2)	
└ Only LAMB	23 (26.4)	37 (41.1)	60 (33.9)	
└ >1 antifungal agent **	20 (22.3)	33 (36.7)	53 (29.9)	0.001
**Treatment strategy (curative)**				
Only amphotericin B	44 (65.7)	20 (35.1)	64 (51.6)	
Only LAMB	23 (34.3)	37 (64.9)	60 (48.4)	0.001
Total (curative therapy)	67 (100)	57 (100)	124 (100)	
Mortality	42 (40.8)	45 (48.4)	87 (43.7)	0.284

* Direct microscopy and culture positivity are reported only among cases with available microbiological data; therefore, comparative statistical analysis with microbiological-negative cases was not applicable. ** More than one antifungal agent refers to sequential or combined antifungal therapy. Antifungal agents are not mutually exclusive because some patients received more than one agent during treatment. NA, not applicable; NS, not statistically significant (*p* ≥ 0.05). The total number of patients varies across variables due to incomplete reporting in the original case reports. Percentages were calculated based on the number of cases with available data for each variable. No data imputation was performed; all analyses were conducted using reported data only. The antifungal agents reported for prophylactic treatment were fluconazole and amphotericin B; the antifungal agents reported for empirical treatment were LAMB, amphotericin B, voriconazole, fluconazole, caspofungin, and ketoconazole; and the antifungal agents reported for curative treatment were LAMB, amphotericin B, posaconazole, fluconazole, itraconazole, and ketoconazole.

**Table 2:** Statistical association between microbiological testing and diagnostic methods.

Diagnostic Modality	Microbiological Examination (199)	Absent n (%)	Present n (%)	Total (n)	χ^2^ (Pearson)	Kappa	Approx. T	*p* Value
**Histopathological findings (n = 188)**	Absent	0 (0)	17 (100)	17	24.16	−0.19	−4.92	**<0.001**
	Present	106 (62.0)	65 (38.0)	171				
**MRI findings**	Absent	63 (56.3)	49 (43.8)	112	0.92	0.07	0.96	0.338
	Present	43 (49.4)	44 (50.6)	87				
**CT findings**	Absent	31 (52.5)	28 (47.5)	59	0.02	−0.01	−0.13	0.894
	Present	75 (53.6)	65 (46.4)	140				
**Radiography findings**	Absent	90 (52.9)	80 (47.1)	170	0.05	−0.01	−0.22	0.824
	Present	16 (55.2)	13 (44.8)	29				
**Clinical examination findings**	Absent	12 (54.5)	10 (45.5)	22	0.02	0.01	0.13	0.899
	Present	94 (53.1)	83 (46.9)	177				
**Symptoms**	Absent	13 (44.8)	16 (55.2)	29	0.97	−0.05	−0.99	0.324
	Present	93 (54.7)	77 (45.3)	170				

MRI: Magnetic resonance imaging, CT: Computer tomography.

**Table 3:** Analysis of the association between mortality and clinical features and treatment approaches in mucormycosis cases.

Variable	Category	Mortality Present n (%)	Mortality Absent n (%)	Total (n)	*p* Value
**Symptoms**	Absent	18 (62.1)	11 (37.9)	29	**0.038**
	Present	69 (41.3)	98 (58.7)	167	
**Mental confusion**	Absent	73 (41.2)	104 (58.8)	177	**0.007**
	Present	14 (73.7)	5 (26.3)	19	
**Hemiparesis**	Absent	66 (40.7)	96 (59.3)	162	**0.025**
	Present	21 (61.8)	13 (38.2)	34	
**Surgical debridement**	Absent	44 (73.3)	16 (26.7)	60	**<0.001**
	Present	43 (31.6)	93 (68.4)	136	
**Posaconazole treatment**	Absent	81 (48.8)	85 (51.2)	166	**0.001**
	Present	4 (14.8)	23 (85.2)	27	
**Liposomal amphotericin B (LAMB)**	Absent	50 (53.2)	44 (46.8)	94	**0.013**
	Present	35 (35.4)	64 (64.6)	99	
**Amphotericin B**	Absent	44 (40.7)	64 (59.3)	108	0.298
	Present	41 (48.2)	44 (51.8)	85	
**Treatment category**	Only amphotericin B	31 (49.2)	32 (50.8)	63	**0.046**
	Only LAMB	26 (43.3)	34 (56.7)	60	
	>1 antifungal agent *	14 (26.9)	38 (73.1)	52	

* Treatment with more than one antifungal agent refers to sequential or combination antifungal therapy. These analyses describe associations and do not imply causality due to the retrospective and descriptive nature of cumulative case-report–based data. LAMB, liposomal amphotericin B.

**Table 4:** Confounder-Adjusted Logistic Regression Analysis of Treatment and Surgical Debridement.

Treatment Variable	Category	n (%)	Crude OR	95% CI for OR	Adjusted OR	95% CI for Adjusted OR	*p* Value
**Posaconazole**	Used	4 (14.8)	0.183	0.060–0.551	0.245	0.019–0.794 *	**0.019**
	Not used	81 (48.8)	Reference	–	Reference	–	
**Liposomal amphotericin B (LAMB)**	Used	35 (35.4)	0.481	0.270–0.858	0.667	0.270–1.271	0.219
	Not used	50 (53.2)	Reference	–	Reference	–	
**Amphotericin B**	Used	41 (48.2)	1.355	0.764–2.404	–	–	
	Not used	44 (40.7)	Reference	–	Reference	–	
**Surgical debridement**	Applied	43 (31.6)	0.168	0.085–0.331	0.180	0.085–0.362	**<0.001**
	Not applied	44 (73.3)	Reference	–	Reference	–	

OR, odds ratio; CI, confidence interval; LAMB, liposomal amphotericin B. * Confidence intervals are wide due to the limited number of events in some treatment categories.

## Data Availability

All data generated or analyzed during this study are included in this published article and its Supplementary Information Files. Additionally, a publicly accessible summary and graphical overview of the findings have been published on our institutional blog: https://mucormycosisturkey.blogspot.com/ (Accessed on 11 June 2026).

## Supplementary Materials

The following supporting information can be downloaded at: https://www.mdpi.com/article/10.3390/jof12060443/s1. Table S1: The CARE checklist questionnaire.
