# Supplementary material for: Overview of Mucormycosis Cases in Türkiye: A Cumulative Case Analysis
Source: J Fungi (Basel). 2026 Jun 17;12(6):443. doi: 10.3390/jof12060443 (PMC13301183; doi:10.3390/jof12060443)
Supplement: Supplementary file 1 [file jof-12-00443-s001.zip › jof-4328907-supplementary.pdf]

CARE Checklist

In this systematic review, 155 articles were assessed according to the CARE Checklist criteria. All the articles (100%) had a structured title, and 135 (87.1%) had an abstract. In all articles (100%), the introduction section was structured, patient information was detailed, clinical findings were clearly given, and diagnostic evaluation was detailed. Treatment and intervention processes were described in detail in 151 articles (97.4%), and follow-up and results were reported in 152 articles (98.1%).

All articles (100%) discussed case contexts in relation to existing literature. However, none included the patient's perspective. Ethical approval was stated in only 28 articles (18.1%), whereas references were cited appropriately in all articles (100%). These findings reveal that case reports generally comply with methodological standards, but important elements such as ethical approval and patient perspective are missing.

Supplementary Table S1**.**The CARE checklist questionnaire

	**Question**	**Sufficient (n)**	**Not Sufficient (n)**	**Partially Sufficient (n)**
1	Does the title clearly reflect the case report?	155	0	0
2	Is a structured abstract provided?	135	7	13

3	Does the introduction explain the context of the case?	155	0	0
4	Is detailed patient information provided?	155	0	0
5	Are clinical findings clearly described?	155	0	0
6	Is the diagnostic evaluation detailed?	155	0	0
7	Are the treatment and interventions clearly stated?	151	4	0
8	Are the follow-up and outcomes described?	152	3	0
9	Does the discussion place the case in the context of the literature?	155	0	0
10	Is the patient perspective provided?	0	155	0
11	Are ethical approval and informed consent included?	27	128	0
12	Are references properly cited?	155	0	0
